# Chicago

**Published:** 1899-09

**Authors:** Joseph B. Clancy

**Affiliations:** Secretary


### SOCIETY PROCEEDINGS.

### CHICAGO VETERINARY SOCIETY.

THE regular monthly meting was called to order by President Robertson, Thursday, June 8th, and in his opening remarks he fittingly voiced the sentiments of the members of the Society by calling attention to the timely opportunity now presented to awaken the dull interest of the people of this city to the great danger that lies in the milk supplied them, more especially the milk from tuberculous cows. Representatives of the press were present, and had given assurance that they would assist in furthering our efforts. The medical society of the city had expressed a willingness to join us in a concerted movement looking toward the suppression of tuberculosis, the inspection of dairies, the testing of cows with tuberculin, and acquire the passage of whatever legislation that may be necessary to make the work thorough and complete. Public interest is now aroused, and the question has presented itself: Who should take the initiative? has naturally and rightfully devolved upon this Society, and no selfish motives should be the inspiration of any member in furthering the work.

Following a spirited discussion, it was resolved, on motion of Dr. E. L. Quitman, that a committee of five be appointed to confer with similar committes representing the medical societies of the city, the joint committee to make the necessary arrangements for a congress on tuberculosis, the subject to be considered at said meeting to be ways and means for the suppression of tuberculosis. The committee was named as follows: Drs. Joseph Hughes, O. E. Dyson, L. A. Merillat, Frank Allen, and R. G. Walker.

Dr. L. A. Merillat presented a paper for discussion, which he said was brief, owing to its numerous subjects.

JOSEPH B. CLANCY,
Secretary.

### DR. MERILLAT'S PAPER ON "SOUNDNESS."

*Fistula or Evidence of Same.* I take this to mean fistula of the withers. A fistula or the tumefaction preceding them is a serious condition, and is therefore sufficient reason for condemning any horse. A recent trauma must always be looked upon with suspicion, as there is no telling how serious a condition may develop from it. As to the scar showing evidence of a pre-existing fistula of the withers, I would not consider that sufficient reason for condemning a horse unless unsightly. Slight scars, together with evidence that the condition had been healed for some time, in fairness to all concerned, might be acceptable. On the other hand, numerous or large cicatrices, with or without tumefaction, should be considered an unsoundness.

*Shoulder-tumors and Galls.* Galls of the shoulders are not to be considered serious unless the conformation of the shoulder is faulty and not suited for the work the animal is required to perform. Conformation of the shoulder is a point never to be overlooked in selecting a horse, especially draught and heavy harness horses. The shoulder should slope evenly from the cartilage of prolongation to the coracoid apophysis, and the collar-seat should be broad and uninterrupted.

Shoulder-tumors are serious in that it requires prolonged idleness to cure them, and are therefor to be considered not sound. No horse having them should be accepted as sound. The loose variety occurring as hyperplastic condition of the dermis, while less painful, are particularly unsightly and are prone to relapse after excision. They are common in mules and in horses in poor flesh. Often animals work without apparent inconvenience for years, but their appearance alone is sufficient reason for condemning the animals.

*Capped Elbow or Shoe-boils* are either hygromata or fibromata. The latter constitute an unsoundness, while the former may be so trivial as to scarcely warrant such a decision. A large hygroma inclosed in a thick wall is serious, of course, and must be dealt with accordingly, and besides it must not be forgotten that these tumors are very prone to recur, because of the difficulty of removing the cause.

*Scars of Meso-neurectomy.* The scar in itself can hardly be considered an unsoundness, but the mere fact that the operation had been performed will lead to the examination for a serious disease below if it had not already been discovered. I can hardly conceive how meso-neurectomy could be performed for any condition not readily recognizable in a glance.

*Broken knees,* which, of course, refer to broken knees that have been healed. I would regard with suspicion any animal with this evidence, as it savors very much of chronic stumbling. If, however, one is satisfied that the animal is not a chronic stumbler but sustained the injury by an unavoidable accident, and the scars are not unsightly, there could be no great harm in passing the horse as sound. They are usually the result of an incurable abnormality of the gait, which in many cases renders an otherwise valuable animal practically useless.

*Speedy Cuts.* The same principle will apply to speedy cuts. If due to unavoidable accident and not to faulty conformation or alteration of normal gait they should be disposed of according to the appearance of the cicatrix.

*Carpitis* is the most serious as well as most common city lameness, and in examination for soundness is dealt with as a lameness by prompt condemnation.

*Synovitis.* With the exception of acute, circumscribed, traumatic synovitis, which will abort in a few days, all animals thus affected must be condemned. This name suggests a variety of special conditions, with locations of equal variance, all of which might be dismissed as very serious conditions from the stand-point of the veterinary examiner.

*Sprain of the Superior Carpal Ligament.* A strain of this structure could not be detected in the absence of lameness.

*Mallenders* is a chronic eczema very refractory to medication or any form of treatment, and is therefore serious and an unsoundness.

*Serous abscesses* are the result of violence, and as they respond promptly to treatment and leave no bad effect might be overlooked under the proper circumstances.

*Discussion.*

Dr. Quitman: I did not quite understand the essayist in regard to shoe-boils. Does not a shoe-boil, even if trivial, attract the attention to the animal's feet? The animal will apparently bring pressure on the back parts of the heel, and will probably bend up the heel to relieve pressure.

I do not believe I am overdrawing when stating that between 60 per cent. and 75 per cent. of all shoe-boils are caused by sore feet.

Dr. Merillat: The querist has evidently misunderstood the paragraph on shoe-boil, so I will re-read it if the Secretary will kindly hand me the manuscript. "Capped elbows or shoe-boils are either hygromata or fibromata. The latter constitute an unsoundness, while the former may be so trivial as to scarcely warrant such a decision. A large hygroma inclosed in a thick wall is, of course, serious, and must be dealt with accordingly, and, besides, it must not be forgotten that these tumors are very prone to recur, because of the difficulty of removing the cause." That is to say, all shoe-boils should be considered sufficient reason for condemning a horse, except the slightest hygromata. And even these should be accepted reluctantly, because of their liability to relapse. Dr. Quitman's argument that 75 per cent. of the animals having shoe-boils also have sore feet is probably true, because such animals lie down a great deal, and are therefore more susceptible, and, besides, horses with contracted feet are apt to be shod with projecting heels, which injure the elbow when the animal is lying down.

Dr. Robertson: Regardng the cause of shoe-boils, the general complaint is that they are due to the mode of shoeing. The writers of our text-books are of the same opinon. I remember when practising with one of the leading practitioners of this city that he frequently complained that the shoers were the cause of them. Commenting on it in particular, I told him I had seen shoe-boils on horses very nicely shod, on horses that had never been shod, and even on the larger breeds of dogs. Certainly these latter did not get them from poor shoeing. I have in mind a stable of horses that are very frequently afflicted with them, and I have traced the cause to the pavement of cedar blocks and poor bedding.

Dr. Merillat: In regard to fistula of the withers, the city veterinarian does not meet with them as frequently as the country practitioner, and as there are members here who have country experience, let us hear their opinion as to the liability of fistulæ to recur.

Dr. Hughes: Being a city practitioner all my life, I have had comparatively little experience with fistulæ of the withers, but the experience I have had has taught me that fistulæ that are once properly healed do not recur. Most of the country practitioners with whom I have discussed the subject are of the same opinion. The question of importance is the cause. It is the most extraordinary thing to find as many as five to fifteen cases occurring simultaneously without any perceptible cause. As to shoe-boils, it is a serious question for the practitioner to decide upon in the examination of horses for soundness. Should a horse with a scar on the elbow or a small nodule the size of a hickory-nut be passed as sound, knowing their liability to recur? It often bothers me to give a decision in these cases. What is the starting point of shoe-boils? Some of our text-books teach us there is a mucous sac at the point of the elbow. Is there such a muco-synovial sac present? Will some one enlighten us on the subject?

Dr. Merillat: A careful dissection of the point of the hock does reveal the presence of a secreting surface, but the same arrangement at the elbow I have always failed to find. The anatomical arrangement and the exposure of the parts to injury should, I think, form the basis of our study as to their cause.

Dr. Allen: I have some statistics on the use of antitoxin for the prevention of tetanus. The stable in which I experimented lost forty-three horses from tetanus during twenty years. During the last year twenty-seven of these died. Since beginning the use of antitoxin, five years ago, I have administered 241 doses in a stable of 624 horses, and have not had a single death from tetanus. On the 1st of last May one of the horses picked up a nail, the teamster did not report, and the horse developed lock-jaw. This was the first case in five years. He had had 10 cc. of antitoxin about eight weeks previously on account of a nail that he had picked up at that time. The case was a very mild one, which I ascribe to the preventive dose administered two months before.

Dr. Quitman: I would like to know of Dr. Allen whether in giving antitoxin he uses antiseptic precautions as well.

Dr. Allen: The foot is pared as usual, and we apply turpentine to the wound, and if there is no lameness the horse is put to work. If lame, we put the foot in a creolin bath.

Dr. Quitman: As a rule, I use all antiseptic precautions, and think it the safest method. This is, of course, not always possible.

Dr. Robertson: My experience with tetanus is that the disease only develops when the wound is nearly healed. Dr. Allen's experiments are certainly sufficient recommendation for the antitoxin treatment, and I have myself found it effectual as a prophylactic measure. While I am aware that many cases recover without treatment, I have seen some very good results from antitoxin as a curative agent.

Dr. Merillat: Dr. Allen's statistics demonstrate two things: The first is that tetanus antitoxin is a good preventive for tetanus, and the second that the barn he mentions needs a thorough disinfection. The street is not the common habitat of tetanus germs; it is the floor of stables that abound with them. If wounds were never exposed to the stable floors it is my opinion there would be fewer cases of tetanus.

Dr. Hughes: I perfectly agree with Dr. Merillat, and would add to his statement that the floors of dark stables are particularly dangerous. I have used 10 cc. of tetanus antitoxin as a preventive, and have found it gives excellent results. As a curative I think it is utterly useless. I have used it in doses as high as 150 cc. without effect.

